# Apparent diffusion coefficient agreement and reliability using different region of interest methods for the evaluation of head and neck cancer post chemo-radiotherapy

**DOI:** 10.1259/dmfr.20200579

**Published:** 2021-05-06

**Authors:** Mustafa Anjari, Amrita Guha, Christian Burd, Marta Varela, Vicky Goh, Steve Connor

**Affiliations:** 1 Department of Radiology, Guy’s and St Thomas’ NHS Foundation Trust, London, UK; 2 School of Biomedical Engineering and Imaging Sciences, King’s College London, London, UK; 3 Department of Radio Diagnosis, Tata Memorial Hospital, Mumbai, India; 4 Homi Bhabha National Institute, Mumbai, India; 5 Neuroradiology Department, King’s College Hospital, London, UK

**Keywords:** Head and neck cancer, Chemoradiotherapy, Diffusion magnetic resonance imaging, Observer variation, Biomarkers

## Abstract

**Objectives:**

Post chemoradiotherapy (CRT) interval changes in apparent diffusion coefficient (ADC) have prognostic value in head and neck squamous cell cancer (HNSCC). The impact of using different region of interest (ROI) methods on interobserver agreement and their ability to reliably detect the changes in the ADC values was assessed.

**Methods:**

Following ethical approval, 25 patients (mean age 59.5 years, 21 male) with stage 3–4 HNSCC undergoing CRT were recruited for this prospective cohort study. Diffusion weighted MRI (DW-MRI) was performed pre-treatment and at 6 and 12 weeks following CRT. Two radiologists independently delineated ROIs using whole volume (ROI_v_), largest area (ROI_a_) or representative area (ROI_r_) methods at primary tumour (*n* = 22) and largest nodal (*n* = 24) locations and recorded the ADC_mean_. When no clear focus of increased DWI signal was evident at follow-up, a standardised ROI was placed (non-measurable or NM). Bland-Altman plots and interclass correlation coefficient (ICC) were assessed. Paired t-tests evaluated interval changes in pre- and post-treatment ADC_mean_ at each location, which were compared to the smallest detectable difference (SDD).

**Results:**

Excellent agreement was obtained for all ROI methods at pre-treatment (ICC 0.94–0.98) and 6-week post-treatment (ICC 0.94–0.98). At 12-week post-treatment, agreement was excellent (ICC 0.91–0.94) apart from ROI_r_ (ICC 0.86) and the NM nodal disease (ICC 0.87). There were significant interval increases in ADC_mean_ between pre-treatment and post-treatment studies, which were greater than the SDD for all ROIs.

**Conclusions:**

ADC_mean_ values can be reproducibly obtained in HNSCC using the different ROI techniques on pre- and post-CRT MRI, and this reliably detects the interval changes.

## Introduction

Advanced head and neck squamous cell carcinoma (HNSCC) is treated by chemoradiation therapy (CRT) at most tumour sites; however, residual disease is still seen at locoregional sites in more than 25% of patients.^1^ Unfortunately, this can be difficult to detect clinically, with post-treatment scarring potentially masking sites of active disease, reducing the window of opportunity for curative surgical treatment. Metabolic imaging with ^18^FluoroDeoxyGlucose positron emission tomography CT (^18^FDG-PET-CT) is widely applied in order to aid the earlier detection of residual disease. An alternative imaging approach is with diffusion-weighted magnetic resonance imaging (DW-MRI), which may combine a qualitative analysis with a quantitative measure of the apparent diffusion coefficient (ADC) for the identification of cellular tumour. In particular, a change in ADC between pre-treatment and post-treatment MRI studies has been proposed as a biomarker for treatment response, with a reduction in cellularity and progressive necrosis resulting in increased ADC values when treatment is successful.^2–4^

There are differing methods used for the acquisition of ADC values with regions of interest (ROIs) being used to sample the whole volume, a maximum cross-sectional area or an area excluding necrosis which is representative of “viable” tumour.^5^

In order for changes in ADC values to be applied to the detection of residual tumour, it must be ensured that the measurements are reproducible and that any variation in ADC values between different observers is less than the interval changes between pre-treatment and post-treatment ADC values. This may be particularly relevant when post-treatment tumour regression obscures the target for ROI placement on the post-treatment MRI sequences.

We aimed to measure the interobserver agreement of ADC_mean_ values recorded at primary tumour and largest pathological nodal locations on the pre-treatment as well as 6-week and 12-week post-CRT DW-MRI in patients with Stage 3–4 HNSCC. Furthermore, the interobserver agreement was calculated for three different methods of obtaining ROIs and the reliability of the ADC_mean_ measurements was assessed for its ability to detect the interval changes in the ADC values.

## Methods and materials

### Patient selection and details

Adult patients were eligible if they had proven Stage 3 or 4 primary HNSCC and *a* ≥ 1 cm^2^ measurable area of disease at the primary or nodal site, and in whom curative primary chemoradiotherapy or radiotherapy (RT) alone was planned. Patients were recruited between May 2014 and May 2015, and staging was performed according to the seventh edition TNM classification for head and neck cancer. Diagnostic biopsies were obtained from the primary tumour (*n* = 21), lymph node (*n* = 3) or both sites (*n* = 1). Patients were excluded if they had prior chemotherapy or RT or evidence of distant metastatic disease.

### MRI

Patients underwent DW-MRI on a 1.5 Tesla Siemens Magnetom Aera system (Siemens Medical Systems GmbH, Erlangen, Germany). Axial echo planar DW-MRI was acquired with multiple b-values (0, 50, 100, 800 and 1500 s/mm^2^) and TR 5900 ms, TE 60 ms, two signal averages, FOV 240 × 240 mm, slice thickness 4 mm with a 4 mm slice gap. Mono-exponential ADC maps were calculated from the *b* = 100 and *b* = 800 values.

### ROI delineation

Two independent radiologists (3 and 7 years experience) delineated ROIs using Osirix v.8.0.2 software on the DWI *b* = 800 s/mm^2^ map, but with access to the post gadolinium fat-saturated T1 axial sequence. ADC_mean_ values were recorded at primary tumour and the largest pathological lymph node locations according to areas demonstrating DWI hyperintensity. By a priori definition, the largest pathological node needed to be >1 cm^2^ in order to be considered as measurable disease. If not clearly pathological on imaging criteria (>1 cm short axis/necrosis/extranodal involvement) then they would have undergone FNA to confirm as pathological. Any areas of necrosis were defined by cross-referencing to areas of either high signal on the *b* = 0 map or absence of gadolinium enhancement.

**Figure 1.**
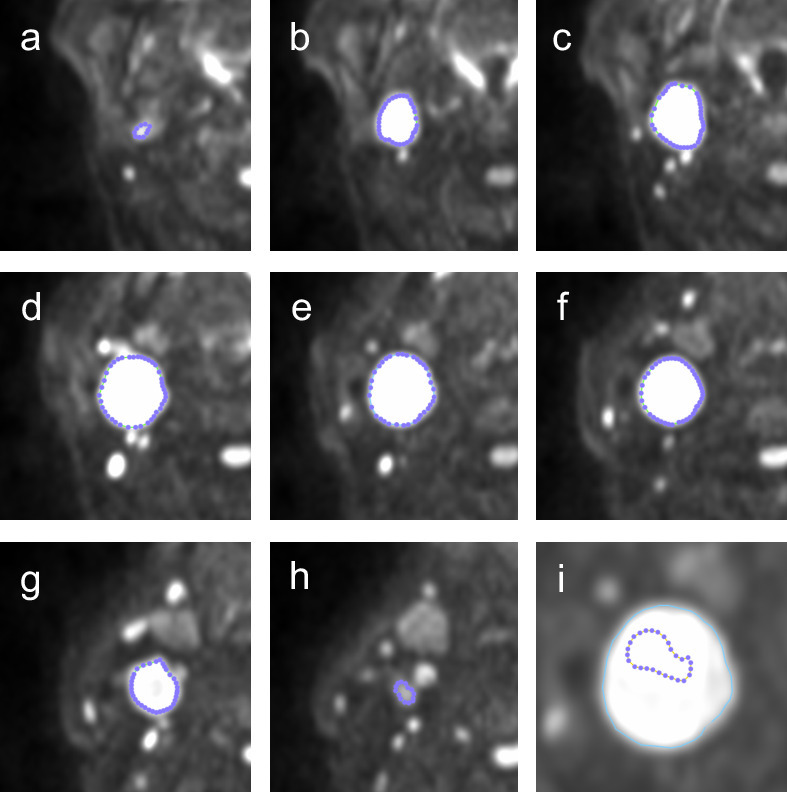
Volumetric ROI (ROI_v_) of the largest lymph node on the b800 image disease was manually delineated (**a–h**) on the b800 DWI sequence. ROI_a_ was analysed on slice e. i is a magnified view of the node in panel e with an example of a representative ROI (ROI_r_). This was focused on an area of increased DWI signal on the b800 image but excluded any areas of necrosis.

**Figure 2.**
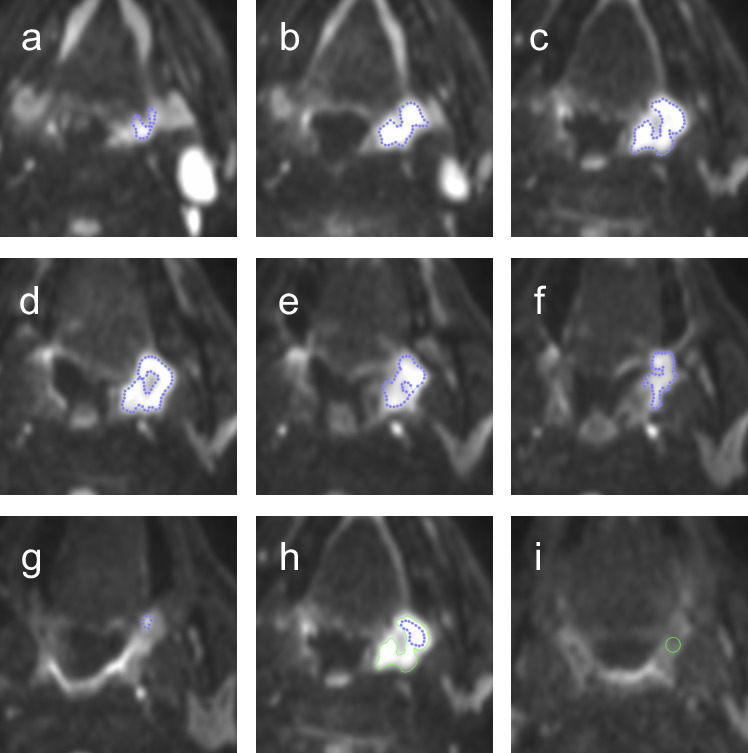
Volumetric ROI (ROI_v_) of a primary HNSCC, and a standardised ROI placed at the site of non-measurable (NM) post-treatment disease. A primary left-sided oropharyngeal tumour is manually delineated on the b800 image (**a–g**). ROI_a_ was recorded from the ROI transferred from slice d. h is a magnified view of the tumour in slice d with an example of a representative area ROI (ROI_r_). There was no measurable (NM) disease at the site of the original tumour at 12-week post-treatment (**i**) so a standardised 6-mm diameter circular ROI (circle) was placed at its original location.

Three freehand separate regions of interest (ROIs) were placed individually within measurable primary tumour and/or largest lymph node on the baseline pre-treatment images:

1. A volumetric ROI placed on multiple sections to encompass the whole of the primary tumour/largest lymph node (ROI_v_, Figures 1a-h and 2a-g).
2. An area ROI placed around the maximum cross-sectional area of the primary tumour/largest lymph node (ROI_a_, Figures 1e and 2d).
3. A representative area ROI placed on the maximum cross-sectional area of the primary tumour/largest lymph node in the core of the lesion (ROI_r_, Figures 1i and 2h). This ROI was focused on an area of increased DWI signal on the *b* = 800 s/mm^2^ map but excluded any areas of necrosis defined by cross-referencing to areas of high signal on *b* = 0 map or the gadolinium enhanced images.

The ROIs were then transferred to the corresponding ADC maps generated from the monoexponential *b* = 100 and *b* = 800 DWIs.

If there was no longer a focus of increased DWI signal on the post-treatment studies to target, a standardised 6-mm diameter circular ROI was placed at its original location (Figure 2i) and this was termed non-measurable (NM) disease. In these cases, it was not possible to perform ROI_v_ and ROI_a_ analysis.

As a reference to assess for measurement stability across timepoints, an ovoid ROI was also placed by one radiologist within the spinal cord at the level of the C2 vertebral body and the ADC_mean_ was recorded.

### Statistical analysis

Interobserver agreement in ADC_mean_ measurements was assessed for pre-treatment, 6-week post-treatment and 12-week post-treatment ROIs using the Bland-Altmann method, as implemented in the Statistics Toolbox in Matlab R2018a (The MathWorks Inc, Natick, Massachusetts, USA). After assessing for data normality using Kolmorogov-Smirnov tests, paired Student’s t-tests were performed. Separate analysis was performed for the post-treatment imaging when termed non-measurable (NM) disease was present. The interobserver intraclass correlation coefficient (ICC) was calculated.

The mean change in ADC between each of the timepoints using all three ROI methodologies was compared with the smallest detectable difference (SDD). This is the smallest difference between interval ADC values that can be interpreted to be “real” when accounting for the potential interobserver variation (based on the agreement statistics). It is, therefore, a conceptually useful result since interval changes larger than the SDD should not simply result from interobserver variation in the measures. The significance level, α, was set to 0.05 and the statistical power, 1-β, to 0.9.

The spinal cord ADC_mean_ data were normally distributed and were compared between different time points with a paired Student’s t-test.

## Results

25 patients (21 male, four female) were recruited with a mean age of 59.5 (range 44.4–73.9) years. There were 18 oropharyngeal (17 HPV positive), four hypopharyngeal and three laryngeal (stage III *n* = 4, stage IVa *n* = 21) tumours. ROIs were delineated at primary tumour (*n* = 22) or largest nodal (*n* = 24) locations. In three of the oropharyngeal (tonsillar) tumours, a primary lesion could not be reliably delineated and one patient with a tongue base tumour had no pre-treatment nodal disease.

At 6 weeks, 20 of 22 (20/22) primary tumours and 10/24 largest lymph nodes were non-measurable. At 12 weeks, 22/22 primary tumours and 19/24 largest lymph nodes were non-measurable. Image artefact precluded accurate ADC_mean_ measurement from ROIs placed at the site of NM primary and nodal disease in one case at 12-week post-treatment, which was excluded from subsequent comparisons. In all comparisons where *n* ≥ 5 in each group, ADC_mean_ values were found to be normally distributed.

**Figure 3.**
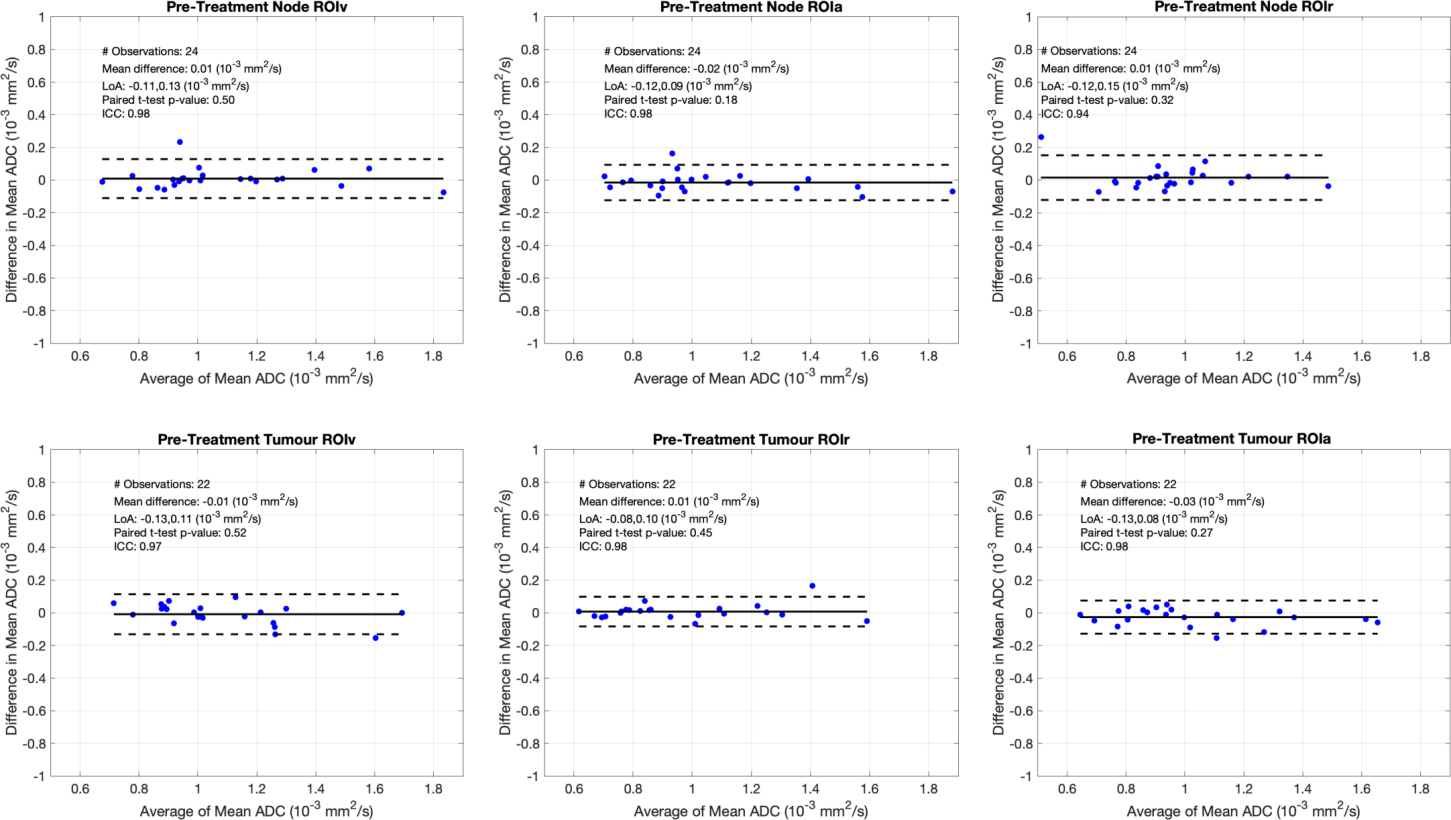
Bland-Altmann plots of ADC_mean_ measurements for ROIs placed within the largest node and the primary tumour on pre-treatment DW-MRI. The horizontal solid line on each plot represents the mean difference in recorded ADC between the two observers, with the hatched lines delineating LoA. Excellent interobserver agreement in ADC_mean_ measurement at baseline at both the site of the primary tumour and within the largest involved lymph node observed across all three ROI methodologies. ICC, Intraclass correlation coefficient; LoA, Limits of agreement.

There was excellent interobserver agreement using all three ROI methods for the pre-treatment evaluation of ADC_mean_ values at nodal and primary tumour locations across all three ROI methodologies (ICC 0.94–0.98) (Table 1 and Figure 3).

**Table 1.** Interobserver comparison of ADC_mean_ measurements in ROIs placed within the largest node or primary tumour at pre-treatment and 6- and 12-week post-treatment DW-MRI

	**Pre-treatment ADC measurements**							
	Node				Tumour			
	ROI_v_	ROI_a_	ROI_r_	NM	ROI_v_	ROI_a_	ROI_r_	NM
Number of observations	24	24	24	–	22	22	22	–
Mean difference (LoA) (× 10^−3^ mm^2^/s)	0.01 (−0.11, 0.13)	−0.02 (−0.12, 0.09)	0.01 (−0.12, 0.15)	–	−0.01 (−0.13, 0.11)	−0.03 (−0.13, 0.08)	0.01 (−0.08, 0.10)	–
ICC	0.98	0.98	0.94	–	0.97	0.98	0.98	–
Paired t-test *p*-value	0.50	0.18	0.32	–	0.52	0.27	0.45	–
	**6-weeks post-treatment ADC measurements**							
	**Node**				**Tumour**			
	**ROI**_**v**_	**ROI**_**a**_	**ROI**_**r**_	**NM**	**ROI**_**v**_	**ROI**_**a**_	**ROI**_**r**_	**NM**
Number of observations	14	14	14	10	2	2	2	20
Mean difference (LoA) (× 10^−3^ mm^2^/s)	−0.03 (−0.17, 0.11)	−0.01 (−0.08, 0.05)	0.00 (−0.10, 0.10)	0.02 (−0.08, 0.12)	N/A	N/A	N/A	−0.01 (−0.20, 0.19)
ICC	0.95	0.98	0.97	0.95	N/A	N/A	N/A	0.94
Paired t-test *p*-value	0.13	0.14	0.81	0.22	0.40	0.10	0.22	0.68
	**12-week post-treatment ADC measurements**							
	**Node**				**Tumour**			
	**ROI**_**v**_	**ROI**_**a**_	**ROI**_**r**_	**NM**	**ROI**_**v**_	**ROI**_**a**_	**ROI**_**r**_	**NM**
Number of observations	5	5	5	18	0	0	0	21
Mean difference (LoA) (× 10^−3^ mm^2^/s)	−0.06 (−0.22, 0.10)	−0.06 (−0.23, 0.12)	0.01 (−0.37, 0.38)	0.01 (−0.21, 0.23)	–	–	–	−0.01 (−0.24, 0.22)
ICC	0.91	0.93	0.86	0.87	–	–	–	0.94
Paired t-test *p*-value	0.17	0.24	0.94	0.78	–	–	–	0.68

ICC, Intraclass correlation coefficient; LoA, Limits of agreement.

Only two patients had persistent tumour at the site of the primary lesion at 6 weeks (and none at 12 weeks) post-treatment, precluding reliable assessment of interobserver agreement in measurable tumoural ADC_mean_ values at this timepoint. Artefact prevented ADC_mean_ measurement at the site of previous disease in one case at 12-week post-treatment.

At 6-week post-treatment, there was excellent agreement for the assessment of the 14/24 cases of measurable and the 10/24 cases of NM nodal disease (ICC 0.95–0.98) as well as the 20/22 cases of NM primary tumour (ICC 0.94) (Table 1 and Supplementary Figure 1).

Supplementary Figure 1.

At 12-week post-treatment, there was excellent interobserver agreement for the ROI_v_ (ICC 0.91) and ROI_a_ (ICC 0.93) analysis of the 5/24 cases of measurable nodal disease, and for the evaluation of the 21/22 NM cases of primary tumour. There was good interobserver agreement for the ROI_r_ (ICC 0.86) analysis of the 5/24 cases of measurable nodal disease and the 18/24 cases of NM (ICC 0.87) nodal disease at 12-week post-treatment (Table 1 and Supplementary Figure 2).

Supplementary Figure 2.

There was no significant difference in values recorded at the site of primary tumour or the largest node between observers using any of the three ROI techniques at any timepoint (paired Student’s t-test *p*-value > 0.10 in all cases).

There was a statistically significant post-treatment increase in ADC_mean_ at both primary and nodal sites from pre-treatment to both 6-week and 12-week post-treatment DW-MRI studies irrespective of the ROI method with a paired/group Student’s t-test *p*-value < 0.002 in all cases (Table 2 and Supplementary Figure 3). These interval increases in ADC_mean_ were larger than the SDD for all ROI methods (Table 2). The ADC_mean_ only increased further between 6- and 12-week post-treatment at sites of NM nodal disease where the interval change also exceeded the SDD.

Supplementary Figure 3.

**Table 2.** Comparison of the mean change in ADC_mean_ between pre-treatment to 6-week post-treatment, pre-treatment to 12-week post-treatment, and 6-week post-treatment to 12-week post-treatment

ADC change between pre-treatment and 6-week post-treatment								
	Node				Tumour			
	ROI_v_	ROI_a_	ROI_r_	ROI_r_/NM	ROI_v_	ROI_a_	ROI_r_	ROI_r_/NM
Mean ∆ADC (× 10^−3^ mm^2^/s)	0.341	0.320	0.413	0.410	0.692	0.753	0.833	0.831
SDD (× 10^−3^ mm^2^/s)	0.259	0.269	0.185	0.185	0.228	0.264	0.262	0.262
Paired/Group t-test *p*-value	**0.001**	**<0.001**	**<0.0001**	**<0.0001**	**<0.0009**	**0.0001**	**0.0002**	**<0.0001**
**ADC change between pre-treatment and 12-week post-treatment**								
	**Node**				**Tumour**			
	**ROI_v_**	**ROI_a_**	**ROI_r_**	**ROI_r_/NM**	**ROI_v_**	**ROI_a_**	**ROI_r_**	**ROI_r_/NM**
Mean ∆ADC (× 10^−3^ mm^2^/s)	0.384	0.470	0.569	0.581	–	–	–	0.896
SDD (× 10^−3^ mm^2^/s)	0.259	0.269	0.185	0.184	–	–	–	0.262
Paired/Group t-test *p*-value	**0.0073**	**0.0028**	**<0.0001**	**<0.0001**	–	–	–	**<0.0001**
**ADC change between 6- and 12-week post-treatment**								
	**Node**				**Tumour**			
	**ROI_v_**	**ROI_a_**	**ROI_r_**	**ROI_r_/NM**	**ROI_v_**	**ROI_a_**	**ROI_r_**	**ROI_r_/NM**
Mean ∆ADC (× 10^−3^ mm^2^/s)	0.043	0.151	0.156	0.207	–	–	–	0.064
SDD (× 10^−3^ mm^2^/s)	0.292	0.245	0.258	0.170	–	–	–	0.317
Paired/Group t-test *p*-value	0.726	0.196	0.238	**0.027**	–	–	–	0.499

Paired/Group Student’s t-tests compared the mean change in ADC_mean_ between the three timepoints using the three different ROI methodologies. Where these changes were statistically significant and larger than the smallest detectible difference (SDD), which accounted for interobserver variation in the measures, the *p*-values are highlighted in bold.

The mean difference in ADC was significantly larger than the SDD for all ROI methodologies between baseline compared to subsequent timepoints. Between 6- and 12-week post-treatment, only ROIs placed at the previous site of primary nodal but now NM disease showed a larger difference in ADC than the SDD.

The spinal cord ADC was normally distributed at each timepoint and there was no significant difference in cord ADC_mean_ between timepoints (baseline *vs* 6-week post-treatment *p* = 0.80, baseline *vs* 12-week post-treatment *p* = 0.07, 6- *vs* 12weeks post-treatment *p* = 0.07).

## Discussion

Our findings indicate excellent interobserver agreement using all three ROI methods for the pre-treatment and 6-week post-treatment evaluation of ADC_mean_ values at nodal and primary tumour locations. The 12-week post-treatment interobserver agreement was also excellent apart from measurable ROI_r_ and NM nodal disease.There were significant changes in ADC_mean_ between 0–6 week and 0–12 week studies, and this was of a magnitude greater than the SDD calculated from the interobserver variation for all ROIs analysed.

Successful CRT treatment results in an increase in HNSCC ADC and when there is a reduction in the expected interval rise in ADC, this is an association with locoregional treatment failure.^2–4^ Such interval changes in tumoural ADC or other diffusion MRI parameters values have been evaluated intratreatment and post-treatment as a prognostic guide.^2–4,6–8^ The majority of studies have found that an increased absolute or ADC_mean_ or a greater rise in ADC_mean_ from pre-treatment to either intratreatment or post-treatment studies, although the finding is not universal. High pre-treatment ADC and low rise in early intratreatment or post-treatment ADC with CRT were consistently observed to be indicators of locoregional failure in the systematic review by Chung et al^4^, although studies were few and heterogeneous. King et al^2^ found that post-treatment ADC showed a significant correlation with locoregional failure at 6 months, with ROC curves determining that an ADC of a post-treatment mass of ≤1.4 ×10^−3^ mm^2^/s achieved 100% specificity and 45% sensitivity for locoregional failure. The optimal threshold of the change in ADC prior to and three weeks following the end of CRT for differentiating responding from non-responding primary HNSCC lesions was found in one paper to be 25%.^3^ In another study, a significant increase in ADC was seen in complete responders within 1 week of CRT treatment for HNSCC, which remained high until the end of the treatment.^6^ This group showed significantly higher increase in ADC than the partial responders by the first week of CRT.

The reproducibility of ADC measurements may vary depending on the technique used to select the ROIs and this impacts on the ability of ADC measurements to detect differences between serial post-treatment studies. Previous data on pre-treatment interobserver agreement in the assessment of ADC values for HNSCC have demonstrated ICC statistics ranging between 0.79 and 0.96.^9–11^ This is potentially influenced by choice of sequence used to define the ROIs, scan parameters, image distortion, choice of nodal versus primary tumour location and lesion size. Our study revealed excellent agreement on pre-treatment evaluation of ADC using all ROI methods at both nodal and primary tumour locations.

To our knowledge, interobserver agreement in ADC measurements following treatment for HNSCC has not been previously assessed; however, this is critical if interval changes are to be used in the evaluation of treatment response. The post-treatment setting creates the added challenge of defining ROIs after regression of a mass lesion, when there may no longer be a clear focal target for the ROI.^12^ When there is no residual post-treatment tumour, it has been described how the ADC may be analysed according to the site of the pre-treatment lesion in studies at other tumour sites.^13^ Our results indicated that such NM disease could also be measured with good or excellent interobserver agreement and that the reliability was sufficient to detect the interval changes in ADC values from pre-treatment to 6- and 12-week post-treatment studies.

The impact on reproducibility of using different ROI methods for the assessment of ADC_mean_ has only been applied to thyroid nodules in the head and neck. There was found to be excellent agreement for malignant thyroid nodules using ROI_a_ and ROI_v_ but only fair agreement for ROI_r_.^14^ This concurs with studies at other tumour sites which have demonstrated decreased interobserver agreement when using smaller ROIs.^15–17^

Our data also indicated the potential for reduction in interobserver agreement with ROI_r_ placed on 12-week post-treatment lymph nodes. This may relate to the additional interpretation required for placing the ROI in an area of solid viable tumour. Rather than assessment based on ADC_mean_ values, Ren et al. investigated the influence of different ROI selection methods on the histogram analysis of ADC maps of locally advanced HNSCC and found decreased agreement when analysing ROI_a_ (ICC 0.51–0.85) compared to ROI_v_ (0.77–0.96).^18^ Whilst our results did not reveal any reduction in the agreement with ROI_a_, it could conceivably introduce bias due to the subjective nature of selecting the appropriate section, which is eliminated by sampling the whole lesion with ROI_v_.

The major limitation of our study is the small sample size and the few patients demonstrating measurable disease following treatment. There were only 5/23 measurable lymph nodes and 0/22 measurable primary tumour at 12-week post-treatment, which precluded adequate assessment of reproducibility in measurable disease post-treatment. This is likely to be due to the preponderance of HPV related oropharyngeal cancer in our study group. This epidemiologically, histologically and clinically distinct form of HNSCC has a well-documented favourable response to CRT. Second, the comparisons were performed exclusively for the mean ADC values and other ADC parameters were not assessed. Although mean ADC values are the most frequent ADC values to be applied clinically, minimum ADC values have also been used for the evaluation of HNSCC. Finally, it is appreciated that there are other factors such as the sequence parameters, the choice and number of b values, the variation in field homogeneity, coil selection between MRI systems^19^ and the postprocessing method which impact on the standardisation and reliability of ADC measurements in HNSCC. All other confounding factors on ADC estimates were set constant in this study in order to determine the exact effect of ROI method and post-treatment effects; however, our results cannot necessarily be translated into other clinical scenarios where they may differ.

## Conclusion

ADC_mean_ values at existing or previous sites of HNSCC can be reproducibly obtained using all three different ROI techniques, even when there is no increased DWI signal as a target. A significant rise in the ADC_mean_ was detected between pre-treatment and either 6-week or 12-week post-treatment studies, and this change was of a greater magnitude than the SDD in measured ADC_mean_ values. This provides further evidence that ADC may be suitable as an imaging biomarker to assess for treatment response between pre-treatment and 6- or 12-week post-treatment MRI studies.
