# Supplementary figures and images for: Apparent diffusion coefficient agreement and reliability using different region of interest methods for the evaluation of head and neck cancer post chemo-radiotherapy
Source: Dentomaxillofac Radiol. 2021 May 6;50(7):20200579. doi: 10.1259/dmfr.20200579 (PMC8474130; doi:10.1259/dmfr.20200579)

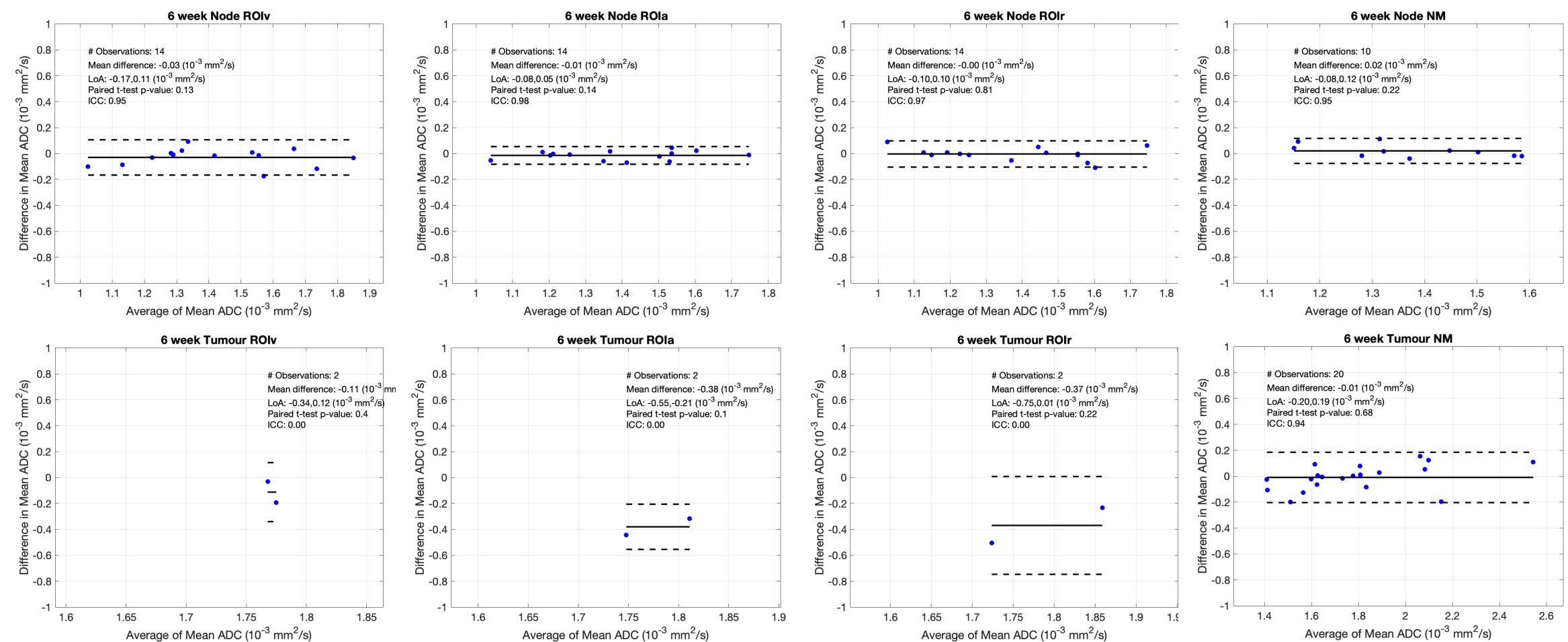

Supplement: Supplementary Figure 1. [file dmfr.20200579.suppl-01.pdf]

12 week Node ROlr

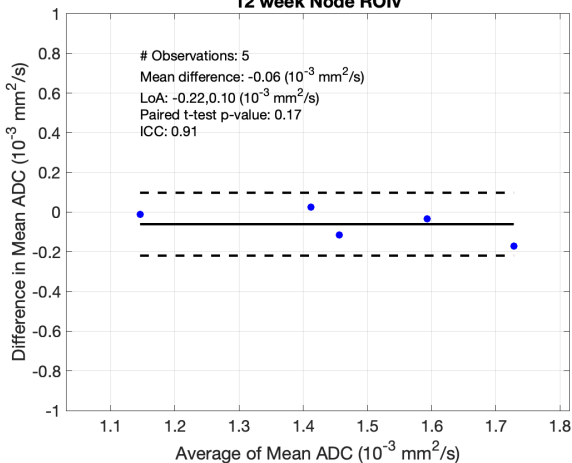

12 week Node ROla

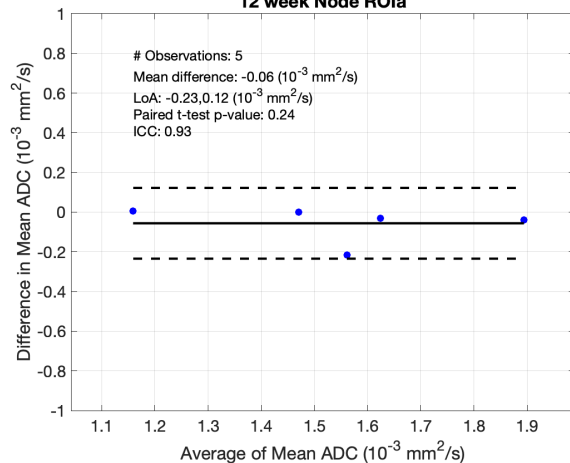

12 week Node ROlr

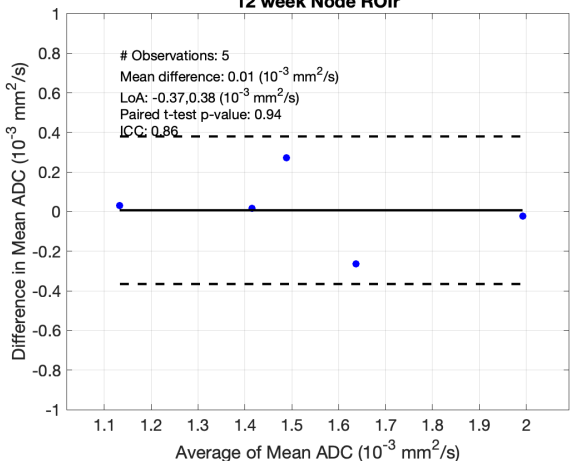

12 week Node NM

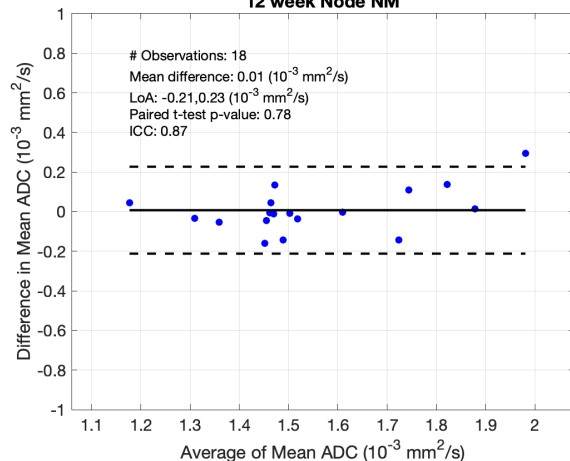

12 week Tumour NM

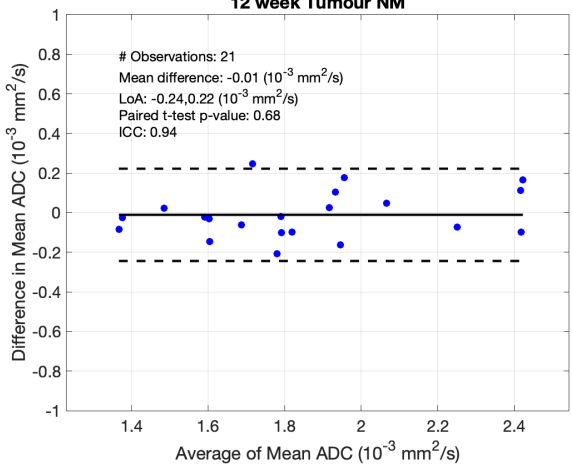

Supplement: Supplementary Figure 2. [file dmfr.20200579.suppl-02.pdf]

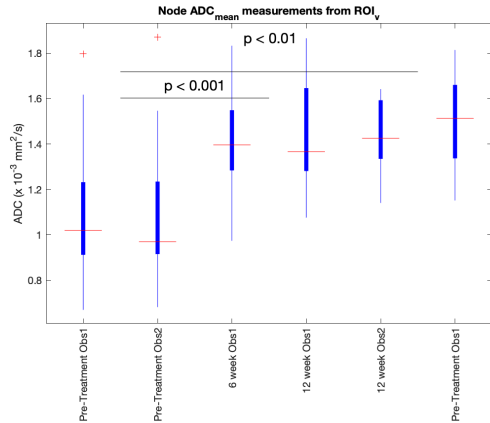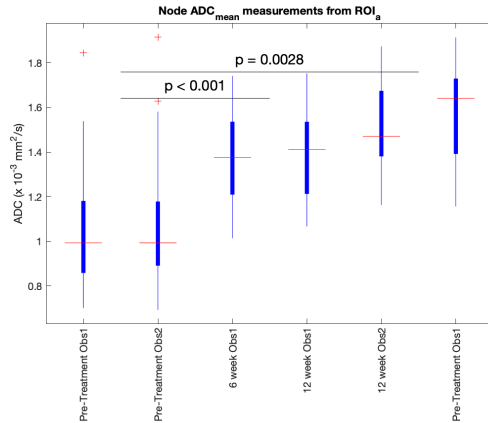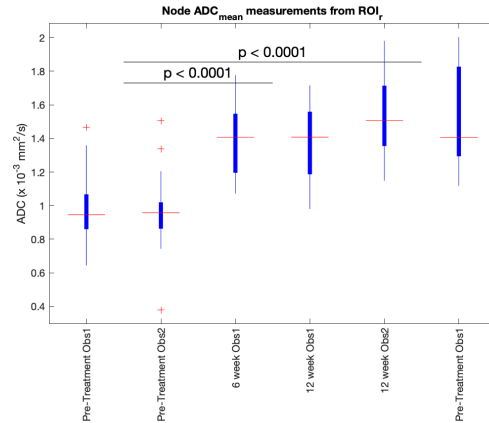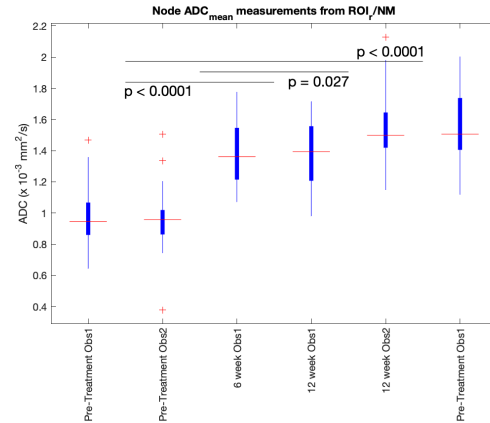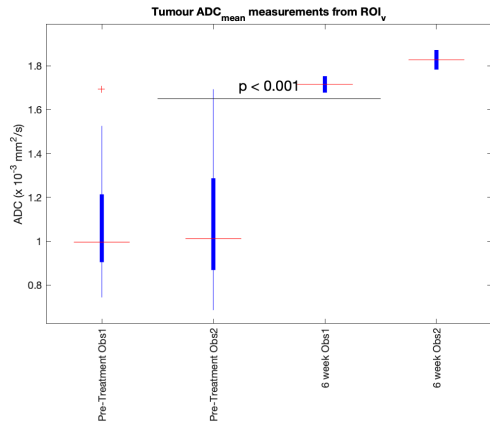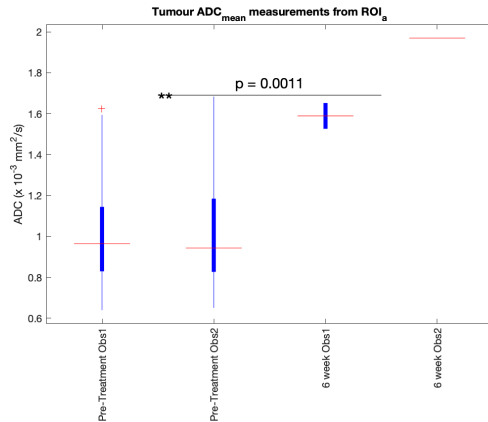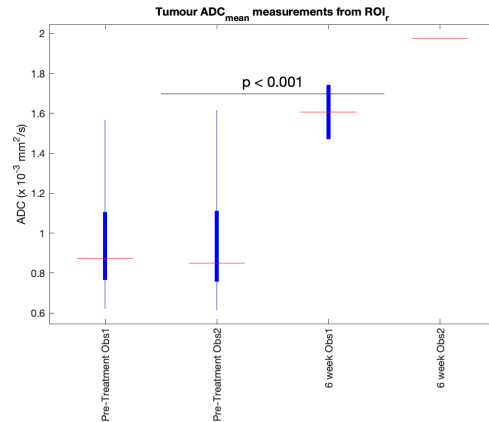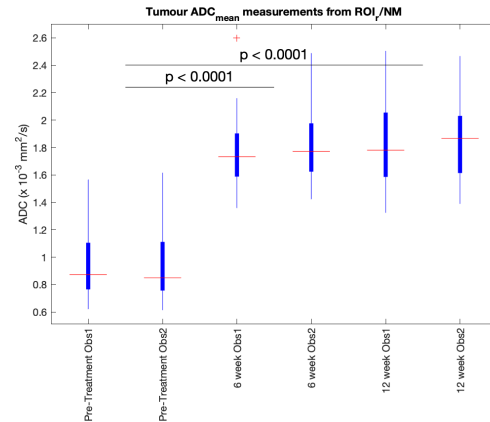

Supplement: Supplementary Figure 3. [file dmfr.20200579.suppl-03.pdf]
